# SiO_2_/H_2_SO_4_: An Efficient Catalytic System for Solvent-free 1, 5-benzodiazepines Synthesis

**Published:** 2012-05-28

**Authors:** Mohammad Reza Shushizadeh, Narges Dalband

**Affiliations:** 1 Research Center of Marine Pharmaceutical Science, Ahvaz Jundishapour University of Medical Science, Ahvaz, IR Iran; 2 Ahvaz Jundishapour University of Medical Sciences, Science and Research Branch- Khoozestan, Ahvaz, IR Iran

**Keywords:** Sulfuric acid, Ketones, 1,2-diaminobenzene, Microwave, Radiotherapy

## Abstract

**Background:**

1, 5-Benzodiazepines have been investigated extensively by organic chemists due to their medicinal and pharmacological properties. These compounds are synthesized by condensation of o-phenylenediamines with carbonyl compounds in the presence of acid catalysts.

**Objectives:**

During our studies on the application of silica resin with acid functional moieties, we found that SiO_2_/H_2_SO_4_ mixture is a simple and efficient catalyst for this method under microwave irradiation.

**Materials and Methods:**

The reaction was carried out simply by grinding SiO_2_/H_2_SO_4_ mixture with o-phenylenediamine, and ketone in the mortar; then the mixture was poured out into a sealed flask. Subsequently, it was irradiated in a microwave oven.

**Results:**

In this method a series of cyclic and acyclic ketones underwent above conversion to form corresponding 1, 5-benzodiazepines.

**Conclusions:**

In conclusion, this method is a simple, rapid, and high yielding reaction (78–95%).

## 1. Background

1,5-Benzodiazepines and their derivatives have been investigated extensively by organic chemists due to their medicinal properties such as analgesic and anti-inflammatory activities (1). These compounds are widely used as anticonvulsant, antianxiety, analgesic, sedative, antidepressive, hypnotic agents as well as anti-inflammatory agents (2). Moreover, 1,5-benzodiazepine derivatives are valuable synthons that can be used in the preparation of other fused ring compounds such as triazolo-, pyrolo-, oxadiazolo-, oxazino-, or furano-benzodiazepines (3). As a result, research in this area is still very active and is directed toward the synthesis of compounds with enhanced pharmacological activity (4).

A new fungus-derived benzodiazepine analogue, 2-hydroxycircumdatin C, has been isolated from a marine natural resource. This compound was isolated from *Aspergillus ochraceus*, an endophytic fungus derived from the marine brown alga *Sargassum kjellmanianum* and displayed significant DPPH radical-scavenging activity (5). Also, a benzodiazepine alkaloid, circumdatin A, was extracted from marine fungus *Cladosporium*. This compound isolated from the surface of the marine red alga *Chondria Crassicualis* and showed moderate antibacterial activity (6). Similarly structured elements among benzodiazepines (BDs) suggest that they might derive from similar building blocks through common biogenetic origins and biosynthetic pathways. Based on the results of feeding experiments, synthesis of BDs requires involvement of several carbonyl compounds and *o*-phenylenediamines.

Generally, these compounds are synthesized by condensation of o-phenylenediamines with a,ß-unsaturated carbonyl compounds (7), ß-haloketones, or ketones (8). A variety of reagents, such as BF_3_-etherate, NaBH_4_, polyphosphoric acid, MgO/POCl_3_, Yb(OTf)_3_, Sc(OTf)_3_, Al_2_O_3_/P_2_O_5_, or AcOH are utilized for this condensation reaction (9). Most recently, this condensation reaction has also been reported to proceed in the presence of CAN, (bromodimethyl) sulfonium bromide, AgNO_3_, NBS, organic acids such as p-nitrobenzoic acid, montmorillonite K10, PPA, 2,4,6-trichloro-1,3,5-triazine (TCT), sulfanilic acid, ZrOCl_2_ , MgBr_2_ and Ga(OTf)_3_ (10-13).

In addition, benzodiazepines were synthesized using green solvents, such as water, ionic liquids and glycerol (14). However, many of these methodologies are associated with several shortcomings like long reaction times, harsh reaction conditions, low product yields, occurrence of several side products, and difficulty in recovery and reusability of the catalysts. Moreover, some of the reagents employed are very expensive. Recently considerable attention has been devoted to heterogeneous organic transformations utilizing inorganic solid acids (15). Some of these compounds such as silica sulfuric acid (SSA) have been employed widely as efficient catalysts for several organic reactions (16). Generally, heterogeneous catalysts offer several advantages including mild reaction conditions, high selectivity, high throughput, and ease of work-up procedures.

Application of microwave energy for conducting organic reactions at highly accelerated rates is an emerging technique. In recent years, microwaves have become popular among synthetic organic chemists for improving classical organic reactions, shortening reaction times, and increasing yields, as well as promoting new reactions (17). Moreover, often when carrying out a reaction in a microwave oven, the use of a solvent can be avoided, which is important in order to make the synthesis more environmentally friendly as it is observed in green chemistry. These observations led us to investigate possibility of improving methods used for solvent-free synthesis of 1,5-benzodiazepine scaffold.

## 2. Objectives

**Figure 1:**
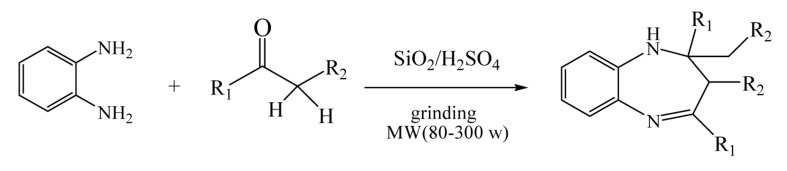
Solvent-free Synthesis of 1, 5-benzodiazepines

During our studies on the application of silica resin with acid functional moieties (18), we found that SiO_2_/H_2_SO_4_ mixture is a simple and efficient catalyst for solvent-free synthesis of 1,5-benzodiazepines under microwave irradiation, as it was followed in (Figure 1).

## 3. Materials and Methods

### 3.1. Reagents and Materials

All starting materials were purchased from Merck and Aldrich companies. The IR spectra were recorded on a Perkin-Elmer RXI infrared spectrometer. ^1^H NMR spectra were recorded by a 400 MHz Broucker FT-NMR spectrometer. TLC accomplished purity of substrates and is contained silica gel polygram SIGL/UV254 plates. The reaction mixture was irradiated in a butane 1000 w microwave oven.

### 3.2. Preparation of SiO_2_/H_2_SO_4_ Mixture

SiO_2_/H_2_SO_4_ mixture was prepared simply in a mortar by grinding 3 g silica gel and 0.5 mL concentrated sulfuric acid for about 5 minutes until a fine and homogeneous mixture was obtained.

### 3.3. General Procedure

The reaction was carried out simply by grinding 0.5 g SiO_2_/H_2_SO_4_ mixture with substrates, to achieve 10 % mole ratio, 10 mmol *o*-phenylenediamine, and 21 mmol ketone in the mortar; , then the mixture was poured out into a sealed flask. Subsequently, it was irradiated in a microwave oven, and reaction was kept to continue for about 5-25 minutes until completion of reaction that was confirmed by TLC monitoring. Then the products were extracted twice by 20 ml diethyl ether. The combined solutions were dried over anhydrous magnesium sulfate. Evaporation of the solvent made the products affordable as seen in Table 1. Structures of products were characterized by their melting points, ^1^H NMR, and IR spectral data.

**Table 1:**
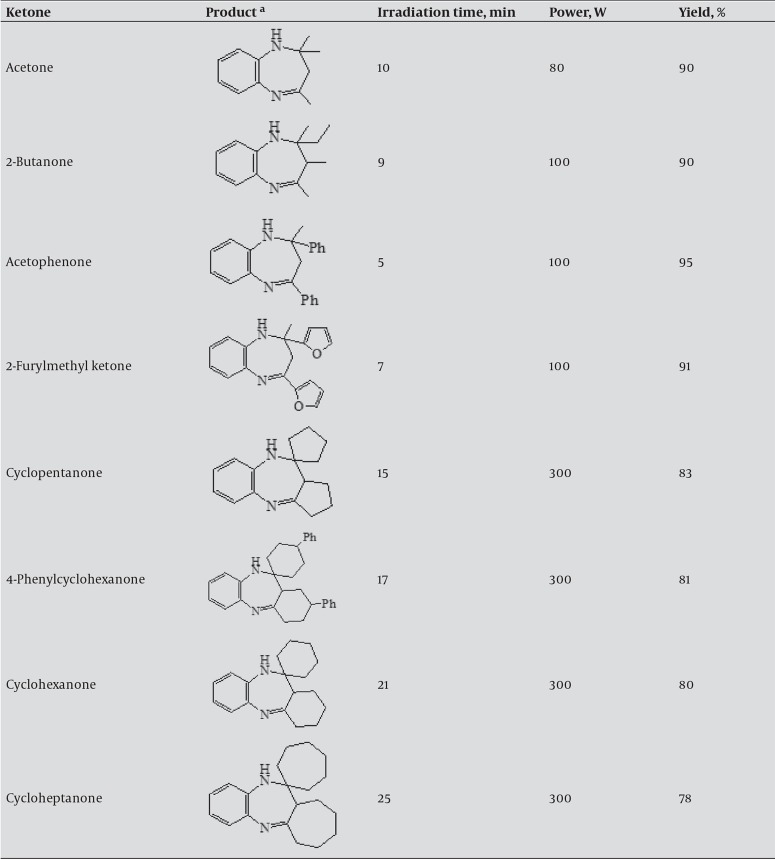
Solvent-free synthesis of 1, 5-benzodiazepines by reaction of o-phenylenediamine (OPD) with various ketones under microwave irradiation ^a^ All products were approved via comparison with authentic samples (IR, 1HNMR, and TLC).

**Figure 2:**
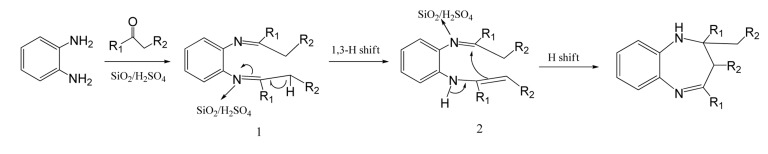
Mechanism for Formation of 1, 5-benzodiazepines

## 4. Results

Table 1 shows a series of cyclic and acyclic ketones underwent above conversion to form a series of 1,5-benzodiazepines. Entries 1-4 in Table 1, e.g. acetone and acetophenone, as aliphatic and aromatic ketones worked well while cyclic ketones, entries 5-8 in Table 1, afforded desired products in lower yields than others. Reaction occurred in a mild condition and experimental procedure was simple. Yield of products was acquired from78% to 95%. Structures of products were determined by their 1H NMR, IR and MS spectral data.

Figure 1 shows a plausible mechanism for 1,5-benzodiazepines formation that involves intermediate Schiff’s base formation, 1, and undergoes 1,3-hydrogen shift to form enamine, 2; intramolecular hydrogen shift provids desired product.

### 4.1. Characterization of Products

Selected spectral data for products shown in Table 1 are given below:

2,2,4-Trimethyl-2,3-dihydro-1H-1,5-benzodiazepine^8^ (entry 1): yellow solid; mp 137–139°C; IR (KBr) cm^-1^: 3330, 1640 (C=N), 1594 (Ar), ^1^HNMR (400 MHz, CDCl_3_, TMS,δ ppm): 6.72–7.14 (m, 4H), 2.97 (bs, 1H, NH), 2.37 (s, 3H), 2.23 (s, 2H), 1.35 (s, 6H).

2-Methyl-2,4-diphenyl-2,3-dihydro-1H-1,5-benzodiazepine^8^ (entry 3): yellow solid; mp 151–152°C; IR (KBr) cm^-1^: 3330, 1635 (C=N), 1594 (Ar), ^1^HNMR (400 MHz, CDCl_3_, TMS,δ ppm): 7.57–7.59 (m, 4H), 7.16–7.31 (m, 7H), 7.03–7.05 (m, 2H), 6.83 (d, 1H), 3.50 (bs, 1H, NH), 3.12 (d, 1H), 2.95 (d, 1H), 1.74 (s, 3H).

Spirocyclopentan-1,2,3,9,10,10a-hexahydrobenzo[b]-cyclopenta[e][1,4]diazepine^8^ (entry 5): yellow solid; mp 138–139°C; IR (KBr) cm^-1^: 3330, 1639 (C=N), 1596 (Ar), ^1^HNMR (400 MHz, CDCl_3_, TMS,δ ppm): 7.32 (dd, 1H), 6.57–7.00 (m, 3H), 3.98 (s, 1H), 2.77 (t, 1H), 2.59–2.63 (m, 2H), 1.92–2.15 (m, 2H), 1.56–1.87 (m, 9H), 1.42–1.48 (m, 1H).

Spirocyclohexan-2,3,4,10,11,11a-hexahydro-1H-dibenzo[b,e][1,4]diazepine^8^ (entry 7): yellow solid; mp 137–139°C; IR (KBr) cm^-1^: 3331, 1637 (C=N), 1590 (Ar), ^1^HNMR (400 MHz, CDCl_3_, TMS,δ ppm): 7.26–7.29 (m, 1H), 6.70–7.00 (m, 3H), 3.78 (bs, 1H, NH), 2.59 (t, 2H), 2.36–2.40 (m, 1H), 1.18–1.86 (m, 16H).

Spirocycloheptane-6, 7,8,9,10,10a,11,12-octahydrobenzo[b]cyclohepta[e][1,4]diazepine^8^ (entry 8): yellow solid; mp 136–137°C; IR (KBr) cm^-1^: 3328, 1641 (C=N), 1590 (Ar), ^1^HNMR (400 MHz, CDCl_3_, TMS,δ ppm): 6.96–7.21 (m, 3H) 6.71–6.73 (m, 1H), 3.59 (bs, 1H, NH), 2.64–2.80 (m, 2H), 2.32–2.36 (m, 1H), 0.99–2.05 (m, 20H).

## 5. Discussions

In conclusion, we have shown that SiO_2_/H_2_SO_4_ mixture is an efficient and simple catalyst for solvent-free synthesis of 1,5-benzodiazepine derivatives the base skeletal backbone for natural products of BDs; it benefits reaction of a wide range of ketones with o-phenylene-diamine under microwave irradiation. Additional eco-friendly attributes of this synthetic protocol are solid state of SiO_2_/H_2_SO_4_, relatively low toxicity, and cost saving.
